# Ecological study on the penetration of induction heating cookers and birth outcomes in Japan

**DOI:** 10.3934/publichealth.2020028

**Published:** 2020-06-05

**Authors:** Yasuto Sato, Kosuke Kiyohara, Sachiko Takehara, Noriko Kojimahara

**Affiliations:** 1 Department of Public Health, School of Medicine, Tokyo Women's Medical University, 8-1 Kawada-cho, Shinjuku-ku, Tokyo 162-8666, Japan; 2 Department of Food Science, Faculty of Home Economics, Otsuma Women's University, 12 Sanban-cho, Chiyoda-ku, Tokyo 102-8357, Japan

**Keywords:** correlation study, electromagnetic field, epidemiology, intermediate frequency, stillbirth

## Abstract

In recent years, equipment that generates intermediate-frequency electromagnetic fields (IF-EMFs) has become increasingly prevalent, and the influence of IF-EMFs on human health is thus attracting increasing attention. The present study was conducted with the aim of analyzing whether there is a relationship between the penetration of induction heating cookers and birth outcomes using an ecological study design at the prefectural level. We created data sets for all 47 prefectures in Japan using previously published statistics. Spontaneous fetal death rate, fetal death rate after 22 weeks of pregnancy, perinatal mortality rate, and proportion of newborns weighing less than 2500 g were used as birth outcomes in correlation analysis. A weak positive association was observed between the penetration of induction heating cookers and the fetal death rate after the 22nd week of pregnancy (r = 0.27, p = 0.07), but it was not statistically significant. In addition, a weak negative association was observed between the penetration of induction heating cookers and the spontaneous fetal death rate (r = −0.27, p = 0.07), but it was not statistically significant. In the present ecological study, no statistically significant association were shown between the penetration of induction heating cookers and birth outcomes. To demonstrate further the safety of induction heating cooker use, observations in epidemiological studies of other designs should be considered.

## 1. Introduction

Intermediate-frequency electromagnetic fields (IF-EMFs) are electromagnetic fields with a frequency of 300 Hz to 10 MHz [1]. In recent years, equipment that generates IF-EMFs has started to become more prevalent in both the home and the workplace, and thus, the influence of IF-EMFs on human health is attracting increasing attention [2],[3]. Sources of pubic IF-EMF exposure include induction heating cookers (cooktops), integrated circuit card readers, anti-theft devices, anti-theft tag demagnetizers, and wireless chargers [4],[5].

Induction heating cookers were first marketed in the 1970s, and have been popularized since around 2000 in Japan [6]. The National Survey of Family Income and Expenditure has been conducted every 5 years since 1959 by the Ministry of Internal Affairs and Communications of Japan, and induction heating cookers have been added as a survey item since 2009 [7]. The average penetration of induction heating cookers for two-or-more-person households nationwide was 18.2% in 2009 and 23.9% in 2014. The penetration of induction heating cookers has thus shown an upward trend in recent years. When cooking using an induction heating cooker, users stand on the side of the equipment. Therefore, it is assumed that persons who use induction heating cookers are exposed to some IF-EMFs (20–90 kHz) [4],[5], especially pregnant women, who often use induction heating cookers at home and whose abdomens protrude to be in a position very close to the device. Given this background, we considered that the influence of IF-EMFs on human health could be observed by investigating the relationship between the penetration of induction heating cookers and birth outcomes.

So far, no health effects from low-level exposure to electromagnetic fields (EMF) have been revealed. On the other hand, in order for safe use of electrical appliances with confidence, it is required to clarify their safety level [2]. For the IF-EMF, some studies with animals and cells have been reported, but the data are limited. Also very few human studies or epidemiological studies have been conducted. It is therefore recommended that further research should be conducted on the health effects of IF-EMF [3]. The present study aimed to analyze the relationship between the penetration of induction heating cookers and birth outcomes using an ecological study design at the prefectural level. This type of study design could be expected to reveal any existing strong associations between the use of induction heating cookers and birth outcomes. We believe that the findings could help establish hypotheses in future epidemiological studies regarding the safety of induction heating cooker use.

## 2. Materials and method

In this study, we created data sets for all 47 prefectures of Japan using previously published statistics. Data on birth outcomes, including the spontaneous fetal death rate (per 1000 births), the fetal death rate after the 22nd week of pregnancy (per 1000 births), the perinatal mortality rate (per 1000 births), and the proportion of newborns weighing less than 2500 g by prefecture in 2009 and 2014 were extracted from the Vital Statistics conducted by the Ministry of Health, Labour and Welfare of Japan (MHLW) [8]. For newborns weighing less than 2500 g, values without multiple births were used. Data on the penetration of induction heating cookers in two- or more person households by prefecture in 2009 and 2014 were extracted from the National Survey of Family Income and Expenditure conducted by the Ministry of Internal Affairs and Communications of Japan [7]. The proportions of smokers among women by prefecture in 2007 and 2013 were extracted from the Comprehensive Survey of Living Conditions conducted by the MHLW [9]. In this study, smokers were defined as those who were over 20 years of age and who had indicated “Smoking every day” or “Smoking on occasion”. The proportion of births at age 35 years or older by prefecture in 2009 and 2014 was calculated from the total number of births and the number of mothers aged 35 years or older. These data were extracted from the Vital Statistics conducted by the MHLW [8]. The numbers of obstetric doctors per 100,000 people by prefecture in 2008 and 2014 were extracted from the Survey of Physicians, Dentists and Pharmacists conducted by the MHLW [10]. The average income per capita by prefecture in 2009 and 2014 was extracted from the Annual Report on National Accounts offered by the Cabinet Office of Japan [11].

Statistical analysis was conducted by creating three data sets: cross-sectional data from 2009 (n = 47), cross-sectional data from 2014 (n = 47), and data on changes from 2009 to 2014 (n = 47). The data on changes from 2009 to 2014 were prepared by subtracting the 2009 values from the 2014 values. Since the present study was an ecological study at the prefectural level, it was difficult to adjust confounding factors by multivariate analysis. Therefore, we analyzed the relationship by using Spearman's rank correlation coefficient. The significance level was set at 0.05. Statistical analyses were performed using R Statistical Software (R Foundation for Statistical Computing, Vienna, Austria).

## 3. Results

Table 1 shows the summary statistics of each variable used in the present study. The mean penetration of induction heating cookers (%) was 18.7 in 2009 and 26.2 in 2014. The mean change from 2009 to 2014 (difference in percentage) was 7.5, showing an upward trend.

**Table 1.** Summary Statistics of Each Variable.

	Mean	Standard deviation	Minimum	Maximum
Spontaneous fetal death rate (per 1000 births)				
2009	11.3	1.46	8.7	15.2
2014	10.8	1.32	8.4	14
Changes from 2009 to 2014	−0.5	1.57	−6.2	2.9
Fetal death rate after the 22nd week of pregnancy (per 1000 births)				
2009	3.3	0.53	2.4	4.3
2014	3	0.61	1.8	4.6
Changes from 2009 to 2014	−0.3	0.65	−1.2	1.3
Perinatal mortality rate (per 1000 births)				
2009	4.2	0.59	2.8	5.4
2014	3.8	0.67	2.2	5.5
Changes from 2009 to 2014	−0.4	0.74	−1.6	1.7
Proportion of newborns weighing less than 2500 g (%)				
2009	8.3	0.68	6.9	10.5
2014	8.3	0.62	6.9	10.3
Changes from 2009 to 2014	0	0.45	−1.1	0.9
Penetration of induction heating cookers (%)				
2009	18.7	6.04	7.3	31.5
2014	26.2	8.44	9.1	44.1
Changes from 2009 to 2014	7.5	4.12	1	18.2
Proportion of smokers among women (%)				
2007	11.2	2.39	7	20.6
2013	9.6	2.19	6.1	17.8
Changes from 2007 to 2013	−1.6	1.29	−5.0	0.1
Proportion of births at age 35 years or older (%)				
2009	20.8	2.49	17	30.1
2014	25.6	2.63	21.2	35.7
Changes from 2009 to 2014	4.8	0.65	3.4	6.1
Number of obstetric doctors (per 100,000 people)				
2008	8.2	1.23	5.7	11
2014	8.7	1.21	6.1	11.6
Changes from 2008 to 2014	0.5	0.57	−0.6	1.9
Average income per capita (1000 yen)				
2009	2552.7	446.97	1945.5	4971.8
2014	2780.3	494.86	2068.2	5406.8
Changes from 2009 to 2014	227.6	112.55	−27.4	538.3

Note: The data to be analyzed were summary statistics for the 47 prefectures of Japan (n = 47). The number of births in 2009 was 1,070,035, and the range of number of births at the prefecture level was 4876 to 106,613. The number of births in 2014 was 1,003,539, and the range of number of births at the prefecture level was 4527 to 110,629. The number of spontaneous fetal death was 12,214 in 2009 and 10,905 in 2014. The number of fetal death after the 22nd week of pregnancy was 3645 in 2009 and 3039 in 2014. The number of perinatal death was 4519 in 2009 and 3750 in 2014. The number of newborns weighing less than 2500 g was 87,281 in 2009 and 81,783 in 2014.

Table 2 shows the results of the correlation analysis for the cross-sectional data in 2009. No significant differences with the penetration of induction heating cookers were observed in the spontaneous fetal death rate (r = −0.24, p = 0.11), fetal death rate after the 22nd week of pregnancy (r = −0.23, p = 0.12), perinatal mortality rate (r = −0.17, p = 0.25), or proportion of newborns weighing less than 2500 g (r = −0.08, p = 0.60).

**Table 2.** Results of correlation analysis with birth outcomes in 2009.

	Spontaneous fetal death rate (per 1000 births)		Fetal death rate after the 22nd week of pregnancy (per 1000 births)		Perinatal mortality rate (per 1000 births)		Proportion of newborns weighing less than 2500 g (%)	
	r	p	r	p	r	p	r	p
Penetration of induction heating cookers (%)	−0.24	0.11	−0.23	0.12	−0.17	0.25	−0.08	0.60
Proportion of smokers among women (%)	−0.00	0.98	0.19	0.19	0.11	0.47	0.00	0.98
Proportion of births at age 35 years or older (%)	−0.11	0.44	0.13	0.40	0.04	0.77	0.16	0.29
Number of obstetric doctors (per 100,000 people)	−0.13	0.38	−0.15	0.32	−0.14	0.35	0.22	0.14
Average income per capita (1000 yen)	−0.22	0.13	−0.09	0.54	−0.15	0.32	−0.08	0.58

Table 3 shows the results of the correlation analysis for the cross-sectional data in 2014. No significant differences with the penetration of induction heating cookers were observed in the spontaneous fetal death rate (r = –0.27, p = 0.07), fetal death rate after the 22nd week of pregnancy (r = −0.08, p = 0.59), perinatal mortality rate (r = −0.12, p = 0.43), or proportion of newborns weighing less than 2500 g (r = −0.25, p = 0.09).

**Table 3.** Results of correlation analysis with birth outcomes in 2014.

	Spontaneous fetal death rate (per 1000 births)		Fetal death rate after the 22nd week of pregnancy (per 1000 births)		Perinatal mortality rate (per 1000 births)		Proportion of newborns weighing less than 2500 g (%)	
	r	p	r	p	r	p	r	p
Penetration of induction heating cookers (%)	−0.27	0.07	−0.08	0.59	−0.12	0.43	−0.25	0.09
Proportion of smokers among women (%)	0.12	0.43	0.07	0.65	0.09	0.57	0.22	0.14
Proportion of births at age 35 years or older (%)	−0.05	0.71	0.00	0.99	0.08	0.62	−0.06	0.68
Number of obstetric doctors (per 100,000 people)	−0.05	0.73	−0.16	0.27	−0.19	0.20	−0.02	0.91
Average income per capita (1000 yen)	−0.25	0.10	0.17	0.25	0.22	0.13	−0.19	0.20

Note: The data to be analyzed were summary statistics for the 47 prefectures of Japan (n = 47). Spearman's rank correlation coefficient and P value were shown.

Table 4 shows the results of the correlation analysis for the data regarding changes from 2009 to 2014. No significant differences with the penetration of induction heating cookers were observed in the spontaneous fetal death rate (r = −0.01, p = 0.96), fetal death rate after the 22nd week of pregnancy (r = 0.27, p = 0.07), perinatal mortality rate (r = 0.21, p = 0.16), or proportion of newborns weighing less than 2500 g (r = 0.04, p = 0.78).

**Table 4.** Results of correlation analysis with birth outcomes using changes from 2009 to 2014.

	Spontaneous fetal death rate (per 1000 births)		Fetal death rate after the 22nd week of pregnancy (per 1000 births)		Perinatal mortality rate (per 1000 births)		Proportion of newborns weighing less than 2500 g (%)	
	r	p	r	p	r	p	r	p
Penetration of induction heating cookers (%)	−0.01	0.96	0.27	0.07	0.21	0.16	0.04	0.78
Proportion of smokers among women (%)	0.08	0.61	0.15	0.33	0.16	0.29	0.01	0.94
Proportion of births at age 35 years or older (%)	0.12	0.43	0.21	0.16	0.29	0.05	0.17	0.24
Number of obstetric doctors (per 100,000 people)	−0.05	0.75	−0.18	0.22	−0.21	0.15	0.03	0.85
Average income per capita (1000 yen)	0.08	0.58	0.09	0.53	0.08	0.61	0.01	0.92

Note: The data to be analyzed were summary statistics for the 47 prefectures of Japan (n = 47). Spearman's rank correlation coefficient and P value were shown.

## 4. Discussion

The present study examined the relationship between the penetration of induction heating cookers (cooktops) and birth outcomes using prefectural data. No statistically significant association were found between the penetration of induction heating cookers and birth outcomes in the present study. However, although not statistically significant, a weak positive association was observed between the penetration of induction heating cookers and the fetal death rate after the 22nd week of pregnancy (r = 0.27, p = 0.07). In addition, a weak negative association was observed between the penetration of induction heating cookers and the spontaneous fetal death rate (r = −0.27, p = 0.07).

Japan is an arc-shaped island country extending about 3000 km north to south and about 3000 km east to west. It has a population of about 127 million and is classified into 47 administrative divisions known as prefectures. The most heavily populated prefectures are Tokyo (13.51 million), Kanagawa (9.12 million), and Osaka (8.83 million), and the most sparsely populated prefectures are Tottori (0.57 million), Shimane (0.69 million), and Kochi (0.72 million) [12]. Although their populations can differ by up to about 20 times, prefectural data reflect the situation of each area, such as living and health conditions. Based on this background, we examined the relationship between the penetration of induction heating cookers and birth outcomes in an ecological study of prefectural units.

In the present study, a weak and not statistically significant positive association was observed in the analysis of the fetal death rate after the 22nd week of pregnancy. In addition, a weak and not statistically significant negative associations were observed in the analysis of the spontaneous fetal death rate. Due to the small sample size (n = 47), the detection power may be insufficient. On the other hand, weak associations in opposite directions were seen in different birth outcomes in the longitudinal and cross-sectional analyses, which were unstable. In addition, the present study conducted repeated statistical tests to analyze multiple birth outcomes. Therefore, it is possible that the observed weak associations were observed by chance. Even if the association was not observed by chance, the following issues can be considered. As for the positive association with the fetal death rate after the 22nd week of pregnancy, because the late stage of pregnancy is known to be stable [8], it is difficult to explain this association biologically. As for the negative associations with the spontaneous fetal death rate, because induction heating cookers are expensive cooking appliances, household income may directly affect the spontaneous fetal death rate.

The limitations of the present study are as follows. Although the differences in the penetration of induction heating cookers among prefectures were large, the differences in birth outcomes among prefectures were small. The data used in the present study were summary statistics for the 47 prefectures of Japan. Therefore, analysis of these data may not be appropriate to detect associations. There is also concern that the sample size in the present study (n = 47) was small. Since this was an ecological study at the prefectural level, it was difficult to adjust confounding factors by multivariate analysis.

The proportion of smokers among women was analyzed since this is known to have an effect on the prevalence of stillbirths [13] and reduced birth weight [14],[15]. The proportion of births at age 35 years or older was analyzed since it is also known to have an effect on the prevalence of stillbirths [13] and reduced birth weight [16],[17]. In addition, the number of obstetric doctors and average income per capita may also affect birth outcomes. However, these factors did not show any significant association in the analysis in the present study. We therefore believe that maternal and child health measures in Japan are intensively implemented [18], and no correlation can be observed at the prefectural level.

## 5. Conclusion

In the present ecological study, no statistically significant association were shown between the penetration of induction heating cookers and birth outcomes. To demonstrate the safety of induction heating cooker use, observations in epidemiological studies using other designs should be considered.
